# Social Cognition in Preschoolers: Effects of Early Experience and Individual Differences

**DOI:** 10.3389/fpsyg.2016.01762

**Published:** 2016-11-14

**Authors:** Daniela Bulgarelli, Paola Molina

**Affiliations:** ^1^Department of Psychology, Università degli Studi di TorinoTorino, Italy; ^2^CHILD, Collegio Carlo AlbertoMoncalieri, Italy

**Keywords:** Theory of Mind, emotion understanding, childcare, language, maternal education, parents’ country of birth

## Abstract

Social cognition is the way in which people process, remember, and use information in social contexts to explain and predict their own behavior and that of others. Children’s social cognition may be influenced by multiple factors, both external and internal to the child. In the current study, two aspects of social cognition were examined: Theory of Mind and Emotion Understanding. The aim of this study was to analyze the effects of type of early care (0–3 years of age), maternal education, parents’ country of birth, and child’s language on the social cognition of 118 Italian preschoolers. To our knowledge, the joint effect of these variables on social cognition has not previously been investigated in the literature. The measures used to collect social cognition and linguistic data were not parent- or teacher-reports, but based on direct assessment of the children through two standardized tests, the Test of Emotion Comprehension and the ToM Storybooks. Relationships among the variables showed a complex pattern. Overall, maternal education and linguistic competence showed a systematic effect on social cognition; the linguistic competence mediated the effect of maternal education. In children who had experienced centre-base care in the first 3 years of life, the effect of maternal education disappeared, supporting the protective role of centre-base care for children with less educated mothers. The children with native and foreign parents did not significantly differ on the social cognition tasks. Limits of the study, possible educational outcomes and future research lines were discussed.

## Introduction

Social cognition is the way in which individuals process, remember, and use information in social contexts to explain and predict how people behave (Fiske and Taylor, 2013). In the current study, two aspects of social cognition were examined: Theory of Mind (ToM) and Emotion Understanding (EU). ToM concerns the attribution of mental states (beliefs, desires, intentions, etc.) to oneself and others, and the ability to use these attributions to understand, predict and explain one’s own behavior and that of other people (Mitchell, 1997). EU, on the other hand, is a component of social cognition and emotional competence, which concerns how individuals understand, predict, and explain their own and others’ emotions (Harris, 1989; Denham, 1998; Saarni, 1999).

From a theoretical point of view, ToM and EU are partly correlated. In Pons and Harris’ (2000) view, EU is made of nine components hierarchically organized. The simplest ones are recognition of emotional expressions and external causes of emotion, followed by the role of desire, beliefs and external reminder on emotions, emotion regulation, displayed emotions, role of moral dimension and mixed emotions. In Wellman’s (1990) approach, basic ToM in childhood consists of five components: recognition of emotion expressions and external causes of emotion, understanding of desire and beliefs, ability to distinguish between physical and mental entities, and awareness of the link between perception and knowledge. Thus, the external features of emotions are necessary to read and predict people’s internal states, while beliefs and desires can shape emotions. The correlation between ToM and EU is also supported by research outcomes (Hughes and Dunn, 1998; Cutting and Dunn, 1999; Pears and Fisher, 2005).

The current study focused on some factors, both external and internal to the child that can influence social cognition abilities in a group of Italian pre-schoolers: the role of early type of care on ToM and EU has been examined together with the effects of other intervening variables as maternal education, parents’ country of birth, and linguistic competence. In what follows, the literature showed that the effect of type of care on social cognition has not been studied yet; that a complex interplay among these factors could be expected and that a study to take into concern simultaneously these several variables is necessary. This study focused firstly on early type of care and other variables that are strictly related to it; some other factors that could influence children’s social cognition development as socio-economic status (Shatz et al., 2003; Cutting and Dunn, 1999; Meins et al., 2013), cognitive functioning and executive functioning (De Stasio et al., 2014; Schneider et al., 2014) were not deepened.

In early childhood, toddlers receive two main types of care: centre-based and home-based. In centre-based care, children experience daily life in a group setting with adults and peers, and routines, spaces and toys are organized for a group of children and adults; in addition, the adults providing the care are trained professionals. In home-based arrangements, children are more likely to be alone with adults or to share routines and toys with a very small number of other children, usually younger or older siblings. In these informal settings, caretakers are usually mothers, grandparents or non-professional baby-sitters (for a broader discussion, see Bulgarelli and Molina, 2016).

The literature emphasizes that type of care is associated with children’s later development, reporting positive effects of centre-based care on cognitive and linguistic outcomes (Broberg et al., 1997; NICHD Early Child Care Research Network, 2002, 2004, 2006; Sylva et al., 2004; Belsky et al., 2007; Loeb et al., 2007; Magnuson et al., 2007; Hansen and Hawkes, 2009). With regard to more general social behavior, centre-based care appears to be related to teacher-reported externalizing problems in preschool and school age children (NICHD Early Child Care Research Network, 2002, 2005). A study on Canadian families showed that maternal care acts as a protective factor in the first year of life as compared to non-maternal care (provided by relatives, non-relatives, day care centres, etc.): parent-reported physical aggression and emotional problems at 4 years of age were lower in children from low-risk families who had been in maternal care (Côté et al., 2008). In the US on the other hand, high quality centre-based care has been found to protect against internalizing and externalizing behavior problems in preschoolers from low-income families (Votruba-Drzal et al., 2004). Thus, besides different types of care have shown to affect cognitive and social dimension of children’s development, as far as we are aware, to date no studies have examined the relationship between social cognition at preschool age and the type of care received during early childhood. Maternal education predicted centre-based care usage in several countries: Norway (Zachrisson et al., 2013), Finland and West Germany (Krapf, 2014), Belgium (Vandenbroeck et al., 2008), UK (Sylva et al., 2007), Italy (Del Boca et al., 2005) and US (NICHD Early Child Care Research Network, 1997a, 2006). Moreover, maternal education is the most robust sociodemographic predictor of mother and infant behavior (Bornstein et al., 2003; Mistry et al., 2008).

Previous research has shown that children’s social-cognitive development is positively associated with parental education level (Perner et al., 1994; Cutting and Dunn, 1999; Pons et al., 2003). In the UK and US, maternal education is positively associated with cognitive and linguistic outcomes (NICHD Early Child Care Research Network, 1997b; NICHD Human Learning Branch, 1998; Peisner-Feinberg et al., 2001; Sammons et al., 2004). Similarly, Italian children’s cognitive and linguistic competence have been found to be systematically related to maternal education (Bulgarelli and Molina, 2016). Moreover, type of care has been shown to moderate the maternal education effect in preschool and school-aged children: specifically, linguistic and cognitive outcomes improve in line with level of maternal education in children who receive home-based care only, indicating that centre-based care can play a protective role in the first 3 years of life (Bulgarelli and Molina, 2016). For this reasons, while deepening the role of early type of care on children’s social cognition, it is crucial to take into consideration the effect of maternal education as well.

Some studies reported that migrant status is related to type of care, specifically by predicting lower utilization of centre-based care (Sammons et al., 2004; Turney and Kao, 2009; Miller et al., 2013, 2014; Zachrisson et al., 2013); though, it is worth noticing that other studies did not find this relationship (Kahn and Greenberg, 2010; Krapf, 2014). A migrant is defined in the United Nations Educational Scientific and Culture Organization Glossary (2016) as “any person who lives temporarily or permanently in a country where he or she was not born, and has acquired some significant social ties to this country”; the parents of first-generation children are both migrants. Social cognition is partly affected by culture (for a review, see Molina et al., 2014), but migrant status is more than a question of cultural belonging: it is a condition with specific features related to entering a new social context–for example, separation from one’s family of origin, changes in economic status, negative stereotypes and discrimination, language barriers and higher levels of stress. Very often, the migrant condition combines with other variables that affect children’s development, such as poverty status and dual language learning, whereby children acquire both their parents’ mother tongue and the language of the host country (De Feyter and Winsler, 2009; Winsler et al., 2014). A Canadian study by Wade et al. (2014) showed that ToM performance at 5 years was predicted by children’s language competence, but not by family income, migrant status or the presence of siblings in the household. Another study by the same research group (Prime et al., 2015) showed that mother’s communicative clarity and mind-reading skills (termed cognitive sensitivity) were positively related to children’s ToM at 5 years, and receptive language and academic achievement at preschool age. This pattern of associations between mothers’ cognitive sensitivity and children’s outcomes was similar in both native and migrant dyads of mothers and children, suggesting that the underlying process was similar. Nevertheless, migrant status appeared to be a risk factor, because it was negatively associated with maternal cognitive sensitivity. In keeping with the findings of Prime et al. (2015), U.S. immigrant mothers have been shown to report higher levels of parenting stress than native mothers, with stress predicting aggressive behavior in pre-school age children (Mistry et al., 2008).

The theoretical frame outlined so far highlighted that the relationship between social cognition development and early type of care requires to focus on other intervening variables, as maternal education and migrant condition, which in turn are related to linguistic issues. Moreover, social cognition and linguistic competence are also “directly” associated with one another. A meta-analysis by Milligan et al. (2007) reported that the predictive correlations between language and ToM were significant, even after controlling for age. When linguistic tasks were administered at an earlier time-point than ToM tasks, the correlations were higher than under the opposite condition, suggesting that the influence of language on ToM is stronger than the influence of ToM on language (Milligan et al., 2007). It may be that an overarching developmental factor such as working memory (Astington and Jenkins, 1999) or executive functioning (Carlson and Moses, 2001) influences both competences. Multiple aspects of linguistic competence may be interrelated with ToM: lexicon (for instance, Lohmann and Tomasello, 2003), syntax (de Villiers and Pyers, 2002) and conversational experience (Harris, 2005; Deleau, 2012). In the literature, debate is ongoing concerning the specific contribution to ToM of the different components of language competence. In the context of this discussion, Miller (2004) has proposed the *performance hypothesis*, which postulates that the influence of linguistic competence on performance on ToM tasks is affected by the linguistic complexity of the ToM task itself; evidence in support of this hypothesis has also come from a study by Bulgarelli and Molina (2013). For a wider discussion of these topics, see Bulgarelli and Molina (2013).

The current study deepens the role of early type of care, maternal education, parents’ country of birth, and children’s linguistic competence on social cognition of a group of Italian pre-schoolers: the reviewed literature showed that a complex interplay among these factors can be expected thus it is worth investigating them together in one study. Moreover, as reported in the Introduction session, previous studies focused on the effect on social behavior: to our knowledge, our study is the first to analyze the role of type of care on ToM and EU. Finally, social behavior was usually measured through parent- or teacher-reported questionnaire (NICHD Early Child Care Research Network, 2002, 2005; Votruba-Drzal et al., 2004; Côté et al., 2008). Parents could be considered reliable observers when they are requested to evaluate children’s behaviors: they have a privileged perspective on their child’s development and can observe the child over time and in a familiar environment (Matheny et al., 1984). Nevertheless, parents are not trained observers: their judgment may be biased by social desirability, they may be incapable of perceiving their children’s real competence (Fenson et al., 1994), and social representation of childhood may play a role in distorting adults’ observations and managing the reliability of the measures (for a wider discussion of this topic, see: Molina and Bulgarelli, 2012b). It is also worth noticing that children’s social cognition involves internal states that are not always directly observable: thus, parents may not be accurate in evaluating this competence (Kårstad et al., 2014). For these reasons, in the current study social cognition was measured directly with the children, through standardized tools that are internationally used to assess ToM and EU.

The current study focused on four research questions, mainly deduced from the literature. The first question related to the effects of type of early childcare on social cognition: given that this was the first study to investigate such question, we relied on earlier findings reported by Bulgarelli and Molina (2016) concerning cognitive outcomes to formulate the second hypothesis, predicting that type of care would only yield an effect in interaction with maternal education: specifically, higher maternal education would positively affect children’s social cognition only in those who had been in home-based care in the first 3 years of life. The second question concerned the role of maternal education on social cognition and we expected that maternal education would directly affect children’s social cognition, in line with the literature reviewed above (Perner et al., 1994; Cutting and Dunn, 1999; Pons et al., 2003). In keeping with the existing literature, the third question concerned the role of parents’ country of birth: no direct effect of this variable on social cognition is expected (Wade et al., 2014; Prime et al., 2015). Finally, the fourth question related to the role of child’s language: in line with earlier studies reported in the literature, as to the fourth hypothesis linguistic competence was expected to be directly associated with social cognition and also to be associated with maternal education (NICHD Early Child Care Research Network, 1997b; NICHD Human Learning Branch, 1998; Peisner-Feinberg et al., 2001; Sammons et al., 2004; Milligan et al., 2007; Bulgarelli and Molina, 2016); we therefore set out to analyze the possible joint effect of maternal education and linguistic competence on social cognition.

## Materials and Methods

### Sample

The sample comprised 118 typically developing children (average age = 59.6 months, *SD* = 10.4, range: 38.5–76.7 months; average *IQ* = 99.6, *SD* = 13.5), all of them attending kindergartens in Turin (Italy): see **Table 1**. Data were collected between 2009 and 2012; most of the children in the current study also took part in earlier reported research by Bulgarelli and Molina (2016).

**Table 1:** Characteristics of the sample.

	Sample	Percentage in the Italian population
**Sample size (%)**	118 (100)	
Male	54 (45.8)	
Female	64 (54.2)	
Average age in years (*SD*, range)	5 (0.9, 3–6)	
Parents’ country of origin (%)		
Both native parents	92 (77.9)	80.6
One foreign parent	14 (11.9)	14.5
Both foreign parents	12 (10.2)	4.9
Mother’s education (%)		
Lower school degree	53 (44.9)	44
Upper school degree	52 (44.1)	41
Higher education	13 (11.0)	15
Type of care (%)		
Home-based	64 (54.2)	76
Centre-based	54 (45.8)	14


Sixty-four children were girls (54.2%). A *t*-test analysis confirmed that the two subsamples of boys and girls were similar with respect to age (*p* = 0.449), IQ (*p* = 0.174), type of early childcare received (*p* = 0.530), maternal education (*p* = 0.187), parents’ country of birth (*p* = 0.650) and verbal quotient (VQ; *p* = 0.450).

With regard to education, 53 mothers had completed lower secondary school (44.9%), 52 held an upper secondary school diploma (44.1%) and 13 were university graduates (11.0%). Overall, the sample displayed a lower level of educational achievement than the Italian population between 25 and 64 years of age in 2011, in which 44% had completed lower secondary education, 41% upper secondary education, and 15% third level education (OECD, 2014). For the purposes of the statistical analysis, the groups of mothers with upper secondary and university-level education were collapsed into one group termed the “highly educated group,” after it had been verified that they did not significantly differ in relation to the independent variables in the research design. A *t*-test analysis confirmed that the two final subsamples of children, with less educated and more highly educated mothers, respectively, were similar in terms of age (*p* = 0.644), gender (*p* = 0.784), type of care (*p* = 0.116) and parents’ country of origin (*p* = 0.163). The IQ and VQ scores of the children with more highly educated mothers were significantly higher than those of the children whose mothers had completed a lower level of education (IQ: *m*_LOW_ = 96.98, *m*_HIGH_ = 101.78, *t*_IQ_ = -1.94, *p* = 0.055; VQ: *m*_LOW_ = 76.70, *m*_HIGH_ = 84.05, *t*_VQ_ = -3.18, *p* = 0.002).

With regard to parent’s country of birth, 92 of the children had two native-born parents (77.9%); 14 had one foreign-born parent (11.9%) and 12 two foreign-born parents (10.2%). In our sample, the percentage of children with two foreign-born parents was slightly lower than in the Italian population (14.5%) and the percentage of children from mixed couples was higher (4.9% in the Italian population; Istat, 2012b). For the purposes of the statistical analysis, the groups of children with two native-born parents and one native-born parent were collapsed into a single subsample labeled native children, after it had been verified that these two groups did not differ in relation to the independent variables in the current research design. A *t*-test analysis confirmed that the two final subsamples, composed of children with at least one native-born parent and first-generation children with two foreign-born parents, respectively, were similar with respect to age (*p* = 0.433), IQ (*p* = 0.104), VQ (*p* = 0.319), gender (*p* = 0.627), type of early childcare (*p* = 0.402) and maternal education (*p* = 0.166).

In relation to type of care, in the first 3 years of life 54 children had received centre-based care (45.8%) and 64 children had been in exclusively home-based care. Home-based care had consisted of either exclusive maternal care or being looked after by other family members or babysitters. In 2010/11, 14.0% of Italian children between 0 and 2 years of age were enrolled in centre-based care, with marked differences among the different geographical regions: for instance, in the North, 29.4% of children attended day care in Emilia Romagna and 15.4% in Piemonte, while in the South, percentages varied from 9.6% in Abruzzo to 2.4% in Calabria (Istat, 2012a). A *t*-test analysis confirmed that the two subsamples of children who had received home-based care and centre-based care were similar with respect to age (*p* = 0.852), IQ (*p* = 0.276), VQ (*p* = 0.136), gender (*p* = 0.530) and parents’ country of birth (*p* = 0.215), but differed significantly in relation to maternal education: highly educated mothers were more likely to choose centre-based care (*p* = 0.021).

### Measures and Procedures

At three separate sessions conducted within a month of each other, the children were individually assessed at kindergarten using four standardized tests: the ToM Storybooks (Molina and Bulgarelli, 2012a; Bulgarelli et al., 2015) were used to assess ToM and the Test of Emotion Comprehension (TEC, Pons and Harris, 2000; Albanese and Molina, 2013) to assess EU; the Leiter-R (Roid and Miller, 2002; US version: 1997) was used to assess non-verbal IQ; the Peabody Picture Vocabulary Test (Dunn and Dunn, 1981), in its Italian version (Stella et al., 2000) was used to assess receptive language, reported as Verbal Quotient (VQ).

The ToM Storybooks is a comprehensive 93-item instrument tapping the five components in Wellman’s (1990) model of ToM: emotion recognition, understanding of desire and beliefs, ability to distinguish between physical and mental entities, and awareness of the link between perception and knowledge; a classical False Belief task is also included. The total score varied from 0 to 111; in this study the total score was used because the standardization of the test is still ongoing. The ToM Storybooks is made up of six full-picture books telling stories about a boy called Sam. Each book recounts an adventure of Sam’s (Sam going to the swimming pool, visiting his grandparents, etc.) and contains 5 or 6 tasks assessing one or more ToM components. The experimenter reads the story while the child looks at the images. In one of the tasks that tap the role of desire in generating behaviors, Sam is searching for his dog: “Where is Puckie? Puckie has hidden [point the picture] behind the tree or [point the picture] behind the trash can. Sam wants to play with Puckie. First, he goes to look behind the trash can. But Puckie is not there. What will Sam do now?”

The TEC evaluates nine hierarchically organized components of EU that emerge between 3 and 11 years. The simplest ones are recognition of emotional expressions and external causes of emotion, followed by the role of desire, beliefs and external reminder on emotions, emotion regulation, displayed emotions, role of moral dimension and mixed emotions. The TEC raw score varied from 0 to 9 and in this study the Italian standardized *z*-score was used. Each TEC components are proposed in the frame of a short pictured story; the child answers to the task questions by indicating the facial expression of the correct emotion, accordingly to what happened. For example, in the displayed emotion task, in which the difference between apparent and real emotion is tapped, the experimenter reads this story: “This is Sarah and this is Dorothy. Dorothy is teasing Sarah because Dorothy has lots of marbles and Sarah doesn’t have any. Sarah is smiling because she doesn’t want to show Dorothy how she is feeling inside. How is Sarah feeling inside? Is she happy, alright, angry or scared?”

Parents were asked to complete a questionnaire on their socio-demographic background, which assessed both parent-related characteristics (country of birth, level of education) and child-related characteristics (country of birth, gender, siblings, type of childcare during the first 3 years of life). Thus, the data concerning the type of early care received in the first 3 years of life was collected retrospectively.

Mothers’ level of education was coded in terms of the Italian school system: (0) lower level of education (i.e., mothers had obtained a low school degree, corresponding to a maximum of 8 years’ school); (1) more highly educated (i.e., mothers had attended at least 13 years of school/university, with high school, bachelor’s, master’s and doctoral degrees all collapsed together into a single category).

For each child, parents’ country of birth was coded as follows: (0) native children (i.e., two native-born parents or one native-born and one foreign-born parent); (1) first-generation children (i.e., two foreign-born parents).

### Analysis

*T*-tests for small sample sizes were performed to check for significant differences in the children’s ToM and EU scores as an effect of type of early childcare, parents’ country of birth and maternal education. The direct effect of language on social cognition was assessed by analyzing the correlations between linguistic competence scores and ToM and EU scores, respectively.

To test for interactions between maternal education and type of early childcare or parents’ country of birth, separate *t*-tests for the effect of maternal education on ToM and EU were performed on the type of care and parental country of origin subsamples. An ANOVA analysis has not been run: the sample size was too small to test the interaction effects through an ANOVA; for this reason, a regression analysis was not run as well.

The mediating effect of linguistic competence was investigated by conducting two regression analyses, with ToM and EU scores as dependent variables and maternal education and parents’ country of birth as independent variables.

## Results

### Direct Effect of Individual Variables

Type of early childcare did not lead to significant differences in ToM and EU and the effect size was not relevant as well (**Table 2**). Maternal education was found to have a significant direct effect on ToM and EU scores. First-generation children obtained the lowest mean scores on the social cognition measures: although these scores did not significantly differ from those of the other children, the effect size was relevant (**Table 2**). Finally, linguistic competence was found to be correlated with both ToM and EU scores (*r* = 0.503, *p* < 0.01 and *r* = 0.406, *p* < 0.01, respectively).

**Table 2:** Differences by type of care, parents’ country of origin and maternal education between groups in relation to ToM and EU average scores (SD).

	*N*	ToM	*t*	*p* (two-tailed)	Cohen’s d	EU	*t*	*p* (two-tailed)	Cohen’s d
**Type of care**									
Home-based	64	54.50 (12.73)	-1.12	0.267	0.21	-0.32 (1.03)	-1.94	0.055	0.36
Centre-based	54	57.70 (17.55)				0.04 (0.98)			
**Parents’ Country of birth**									
Italian-born	106	56.65 (15.29)	-1.47	0.145	0.45	-0.11 (1.01)	-1.36	0.177	0.42
Foreign-born	12	49.92 (12.78)				-0.53 (1.11)			
**Maternal education**									
Low	53	52.92 (13.32)	-2.00	0.048	0.37	-0.42 (1.06)	-2.60	0.010	0.49
High	65	58.45 (16.16)				0.06 (0.94)			


### Interaction among Variables: The Role of Type of Care

Type of care and maternal education were found to interact, in that maternal education had an effect on the social cognition abilities of children who had received home-based care only, but not on those of children who had been in centre-based care. More specifically, children whose mothers had completed a lower level of education only obtained significantly lower ToM scores than children with more highly educated mothers when they had received exclusively home-based care in the first 3 years of life (**Table 3**).

**Table 3:** Differential effects of maternal education as a function of home- versus centre-based early childcare.

Type of care	Maternal education	*N*	ToM (*SD*)	*t*	Cohen’s d	*p* (two-tailed)	EU (*SD*)	*t*	*p* (two-tailed)	Cohen’s d
Home based	Low	33	50.85 (11.82)	-2.46	0.63	0.017	-0.59 (1.06)	-1.74	0.086	0.90
	High	31	58.39 (12.70)				0.03 (0.94)			
Centre based	Low	20	56.35 (15.18)	-0.457	0.12	0.650	-0.14 (1.03)	-0.521	0.605	0.30
	High	34	58.50 (18.97)				0.15 (0.95)			
Total	Low	53	52.92 (13.32)	-2.00	0.37	0.048	-0.42 (1.06)	-2.60	0.010	0.49
	High	65	58.45 (16.16)				0.06 (0.94)			


### Interaction among Variables: The Role of Parent’s Country of Birth

Parental country of birth and maternal education were found to interact: namely, maternal education had an effect on the social cognition abilities of children with native-born parents, but not on those of first-generation children. More specifically, children whose mothers had completed a lower level of education only obtained significantly lower ToM and EU scores than children with more highly educated mothers when both parents were native-born (**Table 4**). Nevertheless, considering the effect size, the differences due to parents’ country of birth were lower than the differences observed in respect to the maternal education.

**Table 4:** Differential effect of maternal education in children as a function of having native-born versus foreign-born parents.

Parents’ country of birth	Maternal education	*N*	ToM (*SD*)	*t*	*p* (two-tailed)	Cohen’s d	EU (*SD*)	*t*	*p* (two-tailed)	Cohen’s d
Native-born	Low	47	53.49 (13.08)	-1.98	0.051	0.38	-0.38 (1.05)	-2.53	0.013	0.49
	High	59	59.17 (16.52)				0.10 (0.93)			
Foreign-born	Low	6	48.50 (15.64)	-0.37	0.720	0.23	-0.71 (1.22)	-0.55	0.597	0.34
	High	6	51.33 (10.48)				-0.35 (1.07)			
Total	Low	53	52.92 (13.32)	-2.00	0.048	0.37	-0.42 (1.06)	-2.60	0.010	0.49
	High	65	58.45 (16.16)				0.06 (0.94)			


### Interaction among Variables: The Role of Linguistic Competence

With regard to the role of linguistic competence, both a direct effect of language on ToM and EU scores and a mediation effect of language on the relationship between maternal education and ToM and EU were found.

**FIGURE 1:**
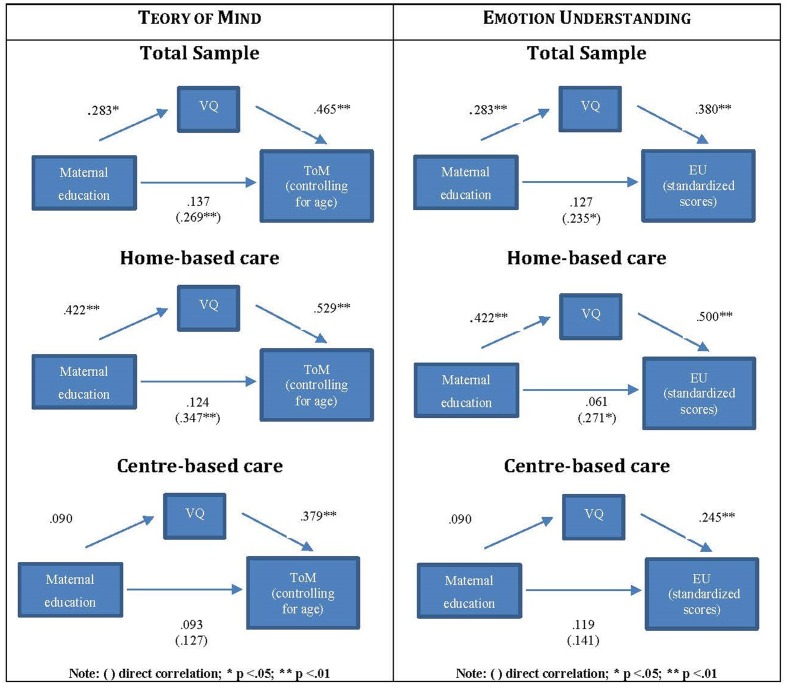
**The mediation of language between maternal education and ToM/EU**.

With respect to ToM (**Figure 1**), the correlation between maternal education and language ability scores was 0.283 (*p* < 0.01), the partial correlation between linguistic competence and ToM scores (after controlling for the effect of maternal education) was 0.465 (*p* < 0.01), while the direct correlation between maternal education and ToM scores was 0.269 (*p* < 0.01), and this correlation was reduced if the language effect was considered (Beta = 0.137, NS).

Turning to EU, the same pattern of results was found (**Figure 1**): the partial correlation between linguistic competence and EU scores (while controlling for the effect of maternal education) was 0.380 (*p* < 0.01), the direct correlation between maternal education and EU scores was 0.235 (*p* < 0.05), and this correlation was reduced if the linguistic competence effect was considered (Beta = 0.127, NS).

When these correlations were analyzed separately in the two groups of children who had received home-based only versus centre-based care, the pattern of results differed (**Figure 1**). With respect to children in home-based care, two significant direct correlations were found: between maternal education and linguistic competence (*r* = 0.422, *p* < 0.01) and between maternal education and ToM scores (*r* = 0.347, *p* < 0.01); furthermore, linguistic competence and ToM scores were correlated, partializing for maternal education (Beta = 0.529, *p* < 0.01). Moreover, linguistic competence mediated the relationship between maternal education and ToM: in fact, the direct correlation between maternal education and ToM was reduced if the linguistic competence effect was considered (Beta = 0.124, NS). With respect to children in centre-based care, the correlation between linguistic competence and ToM was the only significant relationship identified (Beta = 0.379, *p* < 0.01), with no correlations found between maternal education and ToM or between maternal education and language.

A similar pattern of results was found for EU (**Figure 1**): in the subsample of children who had received home-based care only, there were direct correlation between maternal education and linguistic competence (*r* = 0.422, *p* < 0.01), and between maternal education and EU scores (*r* = 0.271, *p* < 0.05); linguistic competence correlated with EU (Beta = 0.500, *p* < 0.01), and mediated the relationship between maternal education and EU: more specifically, the direct correlation between maternal education and ToM was reduced if the linguistic competence effect was taken into account (Beta = 0.061, NS). On the contrary, in children in centre-based care the only significant relationship identified was the correlation between linguistic competence and EU (Beta = 0.245, *p* < 0.01).

When parents’ country of birth was included in the analysis, only linguistic competence was strongly correlated with ToM and EU, in both migrant parent and native-born parent subgroups (**Table 5**). However, no mediation effect was found: parents’ country of birth was not correlated with language (*r* = 0.113, NS for the total sample; *r* = 0.055, NS for children in home-based care; and *r* = 0.154, NS for children in centre-based care), nor with ToM (*r* = 0.101, NS for the total sample; *r* = 0.061, NS for children in home-based care; and *r* = 0.154, NS for children in centre-based care), nor with EU (*r* = 0.125, NS, for the total sample; *r* = 0.071, NS for children in home-based care; and *r* = 0.106, NS for children in centre-based care).

**Table 5:** Correlations among parents’ country of birth, language ability (VQ), ToM Storybooks and TEC scores, after controlling for age.

	*N*	VQ	ToM Scores	EU Scores
Total sample	118			
Parents’ country of birth		0.113	0.101	0.125
VQ		–	0.503^∗∗^	0.406^∗∗^
ToM scores			–	0.347^∗∗^
Native-born parents	106			
VQ		–	0.489^∗∗^	0.380^∗∗^
ToM scores			–	0.307^∗∗^
Foreign-born parents	12			
VQ		–	0.665^∗^	0.820^∗∗^
ToM scores			–	0.598^∗^


*Pearson correlation (Two-tailed): ^∗^*p* < 0.05, ^∗∗^*p* < 0.01.*

## Discussion and Conclusion

The aim of this study was to contribute to the debate about the effects of type of early childcare, maternal education, parents’ country of birth, and child’s linguistic competence on children’s social cognition as observed at preschool age by analyzing Italian data. We analyzed two specific social cognition abilities, ToM and EU, finding them to display a systematically similar pattern of relationships with the independent variables under study.

Interestingly, in our study type of early childcare did not have a direct effect on social cognition and, as predicted according to the first hypothesis, interacted with maternal education: the ToM and EU scores of children who received their early childcare in the home were affected by maternal education, whereas this was not the case for children in centre-based care. It seemed that centre-based care could play a protective role for children with lower-educated mothers: on one hand, professionals provide stimulating contexts and aware educational practice (for a wider discussion, see: Molina, 2016; Molina et al., 2016); on the other hand, in day care services children experience stable and numerous relationship with peers that could foster ToM development: debate is still open about a positive effect of the presence of siblings in the family and peers in kindergarten, observed in some studies (McAlister and Peterson, 2007; Wang and Su, 2009) but not in others (Das and Babu, 2004; Molina and Bulgarelli, 2012a).

According to the second hypothesis, maternal education was found to have a direct effect on ToM and EU: evidence of the effect of maternal education on social cognition has been found in other studies (Perner et al., 1994; Cutting and Dunn, 1999; Pons et al., 2003) as well as in our own earlier study on cognitive outcomes (Bulgarelli and Molina, 2016). A possible explanation for the positive effect of maternal education on children’s development could lay on mothers’ higher awareness of the importance of the quantity and quality of time spent with the offspring: higher-educated parents spend more time with their children than lower-educated parents; they are more aware of the link between spending time with their children and their future development; and are more likely to interiorise and implement the social norms and behaviors associated with “involved parenting” (Sayer et al., 2004; Craig, 2006; Monna and Gauthier, 2008). Moreover, the development of social cognition is specifically supported by parents’ ability to mentalise: mind-mindedness is defined as the adults’ tendency to comment appropriately on their children’s internal states and it plays a protective role for the children’s social development, specifically in low socioecomomic families (Meins et al., 2002, 2013).

In line with the Canadian study by Wade et al. (2014) and the previous Italian study on cognitive outcomes (Bulgarelli and Molina, 2016), an effect of parents’ country of birth on children’s social cognition was not expected. Nevertheless, the results were partly different: no significant differences were found between children with native and foreign parents, but the effect size of the difference between the two groups was not negligible (0.45 and 0.42 for ToM and EU scores, respectively). Then, the lack of significance of the difference could be due to the insufficient power of the statistical test, taking into consideration that the sample was highly unbalanced in favor of children with native parents. Similarly, the very small number of first-generation children could explain the lack of differences due to maternal education observed in this subsample.

As expected based on the literature (for instance, Milligan et al., 2007), the fourth hypothesis was confirmed: linguistic competence directly affected social cognition. On analyzing the role of children’s linguistic competence, which is related to both children’s social cognition and maternal education, linguistic competence was shown to mediate the maternal education effect on social cognition, but only in children in home-based care. As stated before, professional care appeared to play a protective role for children with less educated mothers. The protective role of early type of care was less clear when considering the two groups of children with native and foreign parents: in this case, the linguistic competence seemed the relevant aspect to differentiate children’s performances in the social cognition tasks. In sum, when not correlated with maternal education, language was the variable that mainly correlated with the ToM and EU scores. More highly educated mothers had children with greater linguistic competence, but centre-based care in the early years compensated for this difference. As previously discussed elsewhere (Bulgarelli and Molina, 2016), designing educational intervention and training professionals to better support children’s linguistic development from the early years of life seem crucial: day care services are the context where such support could be better provided (Scopesi and Viterbori, 2008; Molina et al., 2016) and such intervention could be crucial for children with two foreign-born parents.

The role if linguistic competence in shaping the differences among children’s social cognition performances should be interpreted with caution, because children’s performance on the ToM and EU tasks were also affected by the linguistic format of the task itself (Miller, 2004). Moreover, this study focused on receptive language: this measure was chosen because it is a good index of children’s general linguistic competence yet easy and fast to assess; nevertheless, language is a complex construct and future research could deepen the role of other linguistic aspects, as syntax and conversational ability.

With regard to the limits of the current study, the quasi-experimental design required to interpret the results with caution. The sample was recruited in a specific Italian region: this guaranteed a higher homogeneity of social influence on our sample, but limited the generalizability of the results to the Italian population. Italian children’s ToM and EU showed specific pattern of development compared to British and German children (Lecce and Hughes, 2010; Molina et al., 2014): thus the generalizability of the pattern of the current results to other western countries should be specifically tested. Furthermore, the sample included a relatively low number of children with two foreign-born parents, that did not allow to perform a multiple regression analysis to test the interaction of the independent variables; nevertheless, the percentage of this group of subjects was in line with the percentage of children with two foreign parents living in Italy in the period when the data were collected. It is worth noticing that the sample was balanced between medium-low and medium-high socio-economic status, avoiding the biases due to the difficulty in enrolling low socio-economic families. The pattern of the effect of type of early childcare, maternal education, and parents’ country of birth on social cognition was similar to that observed in a previous study in which verbal and cognitive competence were the dependent variables (Bulgarelli and Molina, 2016) and this could be read as a partial support to the validity of the current research. Nevertheless, this pattern of effects might be limited to preschool age, and further investigation with older children is needed. In future research, it would be of interest to explore the role of cognitive functioning and gender in greater depth, together with an index of quality of the type of care in early infancy.

To our knowledge this is the first study to have investigated together the role of early childcare, maternal education, parent’s country of origin and children’s receptive language in the development of social cognition. Type of care, in interaction with maternal education and children’s linguistic competence, affected social cognition and early centre-based care seemed to play a protective role for those children with lower-educated mothers. The protective role of centre-based care was less clear when considering the effect of parental country of birth and further research is needed.

## Ethics Statement

The Comitato di Bioetica dell’Ateneo (the Committee) approved the current research run on human voluntary participants. The Committee approved: 1) the design of the research and the assessment tools; 2) the sample recruitment criteria; 3) the procedure for the collection of the informed consent form. The study involved preschool-aged children: their parents’ consent for participating in the study was collected. The children were observed at school, after an agreement with the school Director and the teachers. Each child gave her or his personal vocal consent to participate in the study assessment.

## Author Contributions

DB and PM substantially contributed to the conception of the work and to the acquisition, analysis, and interpretation of data of the current study. DB and PM wrote the manuscript and revised it critically, adding important intellectual content. DB and PM approved the final version of the manuscript to be published. DB and PM agreed to be accountable for all aspects of the work in ensuring that questions related to the accuracy or integrity of any part of the work are appropriately investigated and resolved.

## Conflict of Interest Statement

The authors declare that the research was conducted in the absence of any commercial or financial relationships that could be construed as a potential conflict of interest.

The reviewer AS and handling Editor declared their shared affiliation, and the handling Editor states that the process nevertheless met the standards of a fair and objective review.
